# Molecular and ecological plant defense responses along an elevational gradient in a boreal ecosystem

**DOI:** 10.1002/ece3.6074

**Published:** 2020-02-05

**Authors:** Rafael Fonseca Benevenuto, Tarald Seldal, James Polashock, Stein R. Moe, Cesar Rodriguez‐Saona, Mark A. K. Gillespie, Stein Joar Hegland

**Affiliations:** ^1^ Faculty of Engineering and Science Western Norway University of Applied Sciences Sogndal Norway; ^2^ Faculty of Environmental Sciences and Natural Resource Management Norwegian University of Life Sciences Ås Norway; ^3^ Genetic Improvement of Fruits and Vegetables Lab Philip E. Marucci Center for Blueberry and Cranberry Research United States Department of Agriculture‐Agricultural Research Service Chatsworth NJ USA; ^4^ Department of Entomology Philip E. Marucci Center for Blueberry and Cranberry Research Rutgers The State University of New Jersey Chatsworth NJ USA

**Keywords:** climate change, constitutive and induced defenses, gene expression, plant‐herbivore interactions, trade‐off

## Abstract

Plants have the capacity to alter their phenotype in response to environmental factors, such as herbivory, a phenomenon called phenotypic plasticity. However, little is known on how plant responses to herbivory are modulated by environmental variation along ecological gradients. To investigate this question, we used bilberry (*Vaccinium myrtillus* L.) plants and an experimental treatment to induce plant defenses (i.e., application of methyl jasmonate; MeJA), to observe ecological responses and gene expression changes along an elevational gradient in a boreal system in western Norway. The gradient included optimal growing conditions for bilberry in this region (ca. 500 m a.s.l.), and the plant's range limits at high (ca. 900 m a.s.l.) and low (100 m a.s.l.) elevations. Across all altitudinal sites, MeJA‐treated plants allocated more resources to herbivory resistance while reducing growth and reproduction than control plants, but this response was more pronounced at the lowest elevation. High‐elevation plants growing under less herbivory pressure but more resource‐limiting conditions exhibited consistently high expression levels of defense genes in both MeJA‐treated and untreated plants at all times, suggesting a constant state of “alert.” These results suggest that plant defense responses at both the molecular and ecological levels are modulated by the combination of climate and herbivory pressure, such that plants under different environmental conditions differentially direct the resources available to specific antiherbivore strategies. Our findings are important for understanding the complex impact of future climate changes on plant–herbivore interactions, as this is a major driver of ecosystem functioning and biodiversity.

## Graphical abstract

We used bilberry (*Vaccinium myrtillus* L.) ramets and an experimental treatment to simulate herbivory by inducing plant defenses (application of methyl jasmonate; MeJA), to observe ecological responses and gene expression along an elevational gradient in a boreal system in western Norway. Across all altitudinal sites, MeJA‐treated plants allocated more resources to herbivory resistance while reducing growth and reproduction, but this response to simulated herbivory was more pronounced at the lowest altitude. These results suggest that plant defense responses at both the molecular and ecological levels are modulated by the combination of climate and herbivory pressure, such that plants under different environmental conditions differentially direct the resources available to specific antiherbivore strategies.

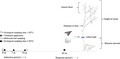

## 1 INTRODUCTION

Over the next century, climate change is projected to considerably alter soil and air temperatures in seasonally snow‐covered temperate forest ecosystems, where high‐latitude tundra and boreal forests are particularly at risk (IPCC, 2018; Serreze et al., 2000). Such environmental changes can have direct and indirect implications for natural ecosystem functioning (Post et al., 2009). For instance, if an increase in temperature dramatically weakens the plant immune system, it could lead to the spread of plant diseases and elevated insect herbivory rates (Cheng et al., 2013; Velásquez, Castroverde, & He, 2018). Also, less snow in high‐latitude ecosystems would result in more mammalian browsing intensity (Danell, Bergstrom, & Iedenius, 1994). Alternatively, plants may adapt to these effects of rising temperatures by investing in effective defense strategies (Bidart‐Bouzat & Imeh‐Nathaniel, 2008). The trade‐offs between such opposing responses and their impacts on plant communities and ecosystem functioning remain poorly understood. In this study, we attempt to address this issue by combining molecular and ecological work across an elevational gradient, and incorporate a methyl jasmonate (MeJA; a ubiquitous defense hormone in plants released in response to stress)‐induced treatment to investigate changes in plant defense responses in the boreal system.

Elevational gradients are “natural experiments” that serve as surrogates for inferring global change‐driven effects (Garibaldi, Kitzberger, & Chaneton, 2011; Körner, 2007; Rasmann, Alvarez, & Pellissier, 2014), because they provide gradual changes in biotic and abiotic conditions along biogeographically tractable scales (Pratt & Mooney, 2013; Rasmann, Alvarez, et al., 2014; Schemske, Mittelbach, Cornell, Sobel, & Roy, 2009). However, our understanding of plant–herbivore interactions is further complicated by the variable defense strategies of some species along elevational gradients, and differential responses to disturbance at the molecular and ecological levels (Moreira, Petry, Mooney, Rasmann, & Abdala‐Roberts, 2018). These challenges can be addressed by studies that combine molecular analysis with ecological studies of induced defenses under natural conditions. Such studies can help us to better understand how abiotic conditions affect plant–herbivore interactions by exploring molecular mechanisms that underpin documented ecological responses. For instance, to cope with environmental variability, plants have evolved the capacity to sense changes in abiotic and biotic factors, quickly reprogram at the molecular level, and adapt through shifts in phenotypic traits (phenotypic plasticity) (Chen, Burke, Velten, & Xin, 2006; Li et al., 2008; Rodríguez, Maiale, Menéndez, & Ruiz, 2009; Winning et al., 2009). Adaptive strategies to biotic and abiotic stress are coordinated by cellular and molecular activities aiming to minimize damage and, at the same time, conserve valuable resources for growth and reproduction (Ahuja, Vos, Bones, & Hall, 2010). Recently, the use of “omic” approaches has been effective in identifying molecular changes at transcript, protein, and metabolite levels that confer resistance or are regulated in response to environmental stressors (Bokhari, Wan, Yi‐Wei Yang, Zhou, & Liu, 2007; Springer, Orozco, Kelly, & Ward, 2008; Zeller et al., 2009; Zobayed, Afreen, & Kozai, 2005). Variation in gene expression, and consequently synthesis of its functional products (i.e., proteins and metabolites), is known to play a role in the evolutionary processes of natural populations in response to environmental adaptation (Oleksiak, Churchill, & Crawford, 2002; Schadt et al., 2003). Whether these effects of adaptive plasticity, alone or in combination, can affect ecosystem functioning depends on how such molecular and phenotypical changes influence trophic interactions (Hegland, Nielsen, Lázaro, Bjerknes, & Totland, 2009).

Bilberry (*Vaccinium myrtillus* L.) is a long‐lived dwarf shrub that provides an excellent model organism for combined molecular and ecological study. The plant is considered as a key food source for herbivores, pollinators, and fruit eating birds and mammals in northern European boreal forest ecosystems (Hegland et al., 2009; Hjältén, Danell, & Ericson, 2004; Jacquemart, 1993; Selas, 2001), and has been found to be sensitive to environmental changes. In previous studies, we showed that defenses induced by herbivore feeding or treatment with MeJA reduce herbivory and increase reproduction of the damaged or treated plants (Benevenuto et al., 2018; Hegland, Seldal, Lilleeng, & Rydgren, 2016; Seldal, Hegland, Rydgren, Rodriguez‐Saona, & Töpper, 2017). These studies provide evidence of potential trade‐offs between growth and defense in bilberry plants when coping with herbivore attack. Additionally, MeJA‐induced bilberry plants were shown to exhibit multiannual effects on herbivore resistance (Benevenuto et al., 2018). However, it is still unclear whether such trade‐offs between defense strategies (i.e., constitutive or induced) and growth and reproduction are modulated by changes in environmental conditions.

Using wild bilberry as a model organism and MeJA application to simulate the effects of herbivory, we investigated the effects of the treatment on plant defense responses, in terms of gene expression and ecological traits, across an elevational gradient and over two consecutive years. The gradient used in this study included optimal growing conditions for bilberry at a “medium” elevation (ca. 500 m a.s.l.), and the plant's range limits in this region at high (ca. 900 m a.s.l.) and low (ca. 100 m a.s.l.) elevations. We expected MeJA‐treated plants to allocate resources from growth and reproduction to defenses at the molecular (i.e., upregulation of defense genes and downregulation of genes related to photosynthesis/nitrogen metabolism) and ecological levels (i.e., reduction of growth, reproduction, and consequently herbivory). As induced defenses are presumed to be energetically costly (Benevenuto et al., 2018; Karban, Yang, & Edwards, 2014; Nabity, Zavala, & DeLucia, 2013; Rodriguez‐Saona, Polashock, & Malo, 2013; Seldal et al., 2017) and decline with increasing elevation (Moreira et al., 2018; Pellissier et al., 2016), we predicted that this effect would be most pronounced at the lowest elevation (*Prediction I*). According to the “optimal defense theory” hypothesis, plants at lower elevations should invest more on defenses due to high risk of herbivory (Rhoades, 1979). Alternatively, as bilberry is a slow‐growing species adapted to limiting abiotic conditions (Flower‐Ellis, 1971), its defense system should be affected by environmental changes (Bidart‐Bouzat & Imeh‐Nathaniel, 2008; DeLucia, Nabity, Zavala, & Berenbaum, 2012; Veteli, Kuokkanen, Julkunen‐Tiito, Kuokkanen, Julkunen‐Tiito, Roininen, & Tahvanainen, 2002). Based on the “resource availability hypothesis,” plants should constantly invest in defenses when growing under low resource availability (Coley, Bryant, Stuart, & Chapin, 1985). If the resource availability hypothesis is true, we expect plants from the high elevation to be under constant state of “alert,” which could lead to high investment in constitutive defenses (Moreira et al., 2014; Pellissier et al., 2016), resulting in consistently reduced seasonal growth and reproduction and decreased herbivory rates, as well as higher basal expression levels of defense genes compared to plants at the optimum and low elevations (*Prediction II*).

## 2 MATERIALS AND METHODS

### 2.1 Study system

The study was conducted within a pine bilberry forest ecosystem in Kaupanger, inner Sognefjord, western Norway (61.2°N, 007.2°E), between May/June and August/September of 2016 and 2017. The area has annual precipitation of 700–900 mm and a mean summer temperature range of 12–16°C (Moen, 1999). The inner part of Sognefjord offers marked topographical variation, representing elevational gradients of 0–2,400 m above sea level (m a.s.l.) over horizontal distances of about 2 km, which makes it a practical and convenient location for gradient‐based field experiments. The most abundant vascular plant species in the field layer are bilberry, lingonberry (*Vaccinium vitis‐idaea* L.), and crowberry (*Empetrum nigrum* L.), while Scots pine (*Pinus sylvestris* L.) and birch (*Betula pendula* Roth and *B. pubescens* Ehrh.) dominate the tree layer. The area has a dense winter population of red deer (*Cervus elaphus* L.) (S. J. Hegland, *personal observation*), which is the most abundant wild ungulate in Norway (Norwegian Environment Agency, 2017).

Bilberry, our study species, is a long‐lived deciduous clonal dwarf shrub, with evergreen stems generally 10–60 cm high (Flower‐Ellis, 1971; Ritchie, 1956). Although we do not have specific information regarding clone size and distribution for the study area, we have based our work on the assumption that rhizomes can reach around 200 cm in length, depending on age (Flower‐Ellis, 1971), and the proportion of genetic variation within population is high (Albert, Raspé, & Jacquemart, 2003, 2004). Bilberry is considered a key species in the boreal ecosystem because of its ecological role as food source for many vertebrate and invertebrate herbivores (Hegland, Jongejans, & Rydgren, 2010). The main mammalian herbivores feeding on the plant in the study area are red deer and various rodent species (Hegland et al., 2016), whereas the most common insect herbivores are larvae in the family Geometridae (Atlegrim, 1989). Bumblebees, honeybees, and syrphid flies are the main pollinators of bilberry (Jacquemart, 1993; Jacquemart & Thompson, 1996).

### 2.2 Study design

**Figure 1:**
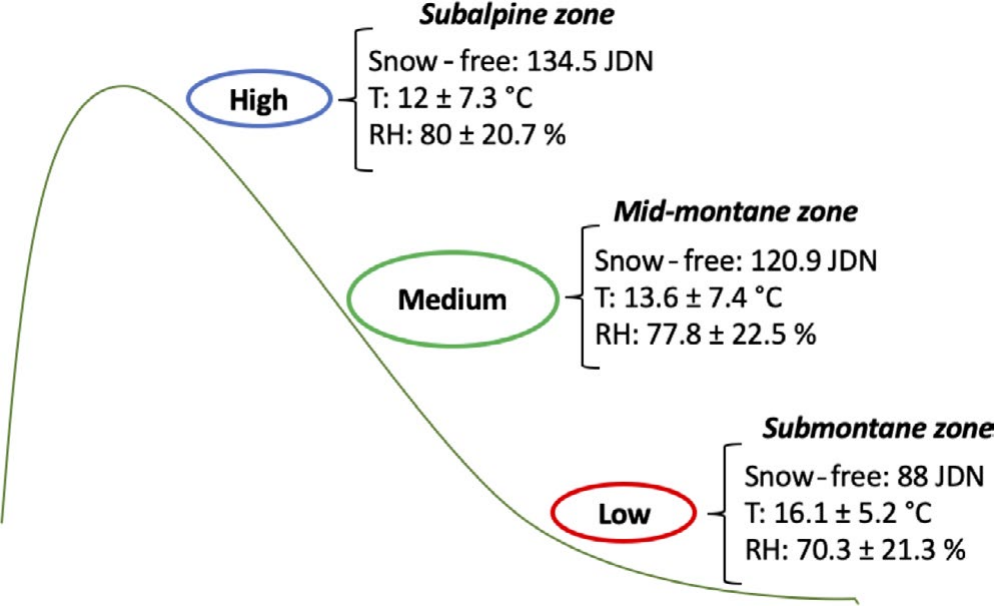
Average timing of snow melt, temperature, and relative humidity across the elevational gradient. Snow‐free = Julian day number (JDN) for complete snow melt; T = air temperature; and RH = relative air humidity

A natural elevational gradient ranging from just above sea level to the tree line was used to investigate the effects of variation in environmental conditions, such as temperature and timing of snow melt, on plant defense responses at the molecular and ecological levels. The experiment was conducted at three different elevational sites: “Low” = ca. 100 m a.s.l. (submontane zone); “Medium” = ca. 500 m a.s.l. (mid‐montane zone); and “High” = ca. 900 m a.s.l. (subalpine zone) (Figure 1). Data on average temperature and relative humidity were obtained from four data loggers (TRIX 8 LogTag, Auckland, New Zealand) placed at each site during the entire study season (June through September). Timing of snow melt was recorded as the Julian day on which each site became snow‐free (Figure 1). This gradient covers a relatively large part of the climatic distribution of the species, from temperate to alpine areas (Moen, 1999). At all elevations, the experiment was established in sites with similar characteristics, including the same south‐facing slopes (16.4 ± 8.0 [*SD*] degrees on average), and similar light availability among sites. Low and Medium sites consisted of more than 15‐year‐old clear cuts with relatively low abundances of small pines and birches that produced negligible shadow effects. The High site was a naturally open subalpine area just below the tree line. Vegetation structure was similar among the sites, with a field layer and scattered tree layer up to ca. 5 m. Unpublished data from 2016 showed that there was relatively little variation in soil pH (mean, 4.19), while soil organic matter (mean, 60.4%) was slightly lower at the low elevation (Knut Rydgren *Personal Communication*, 2016).

In May/June of 2016, 20 blocks were established at each site measuring 150 m^2^ (10 m × 15 m). Within each block, two ramets (between 10–25 cm height) at least 10 m apart were randomly selected (*N* = 120 plants), and each was exposed to one of two different treatments following the same methodology as previous studies (Benevenuto et al., 2018; Seldal et al., 2017): (a) 10 mM MeJA application (treated plants), and (b) water/ethanol application (control plants). Several studies have shown that inducible defense responses in plants can be activated by exogenous application of MeJA (Baldwin, 1999; Benevenuto et al., 2018; Hegland et al., 2016; Van Dam & Baldwin, 2001; Yang, Huihui, Xie, & Rantala, 2013). To achieve the desired concentration of MeJA, 4.1 M MeJA stock (Bedoukian Research, Danbury, CT) was diluted 1:10 with 95% (v/v) ethanol and rediluted with water to get a final concentration of 10 mM MeJA (Seldal et al., 2017). Ethanol was added to water at the same final concentration as that in the 10‐mM MeJA solution (41:1) for the control. To avoid rapid evaporation of MeJA, a cotton wad was attached to the stem at the ground and saturated with 10 mM MeJA or water/ethanol (control) until the point of runoff. Applications were repeated three times with 1‐week intervals to induce the plant defense response associated with an attack by herbivores. The plants were only exposed to treatments in 2016 to evaluate possible multiannual effects of the MeJA induction treatment on bilberries growth and defenses.

### 2.3 Sampling procedure

**Figure 2:**
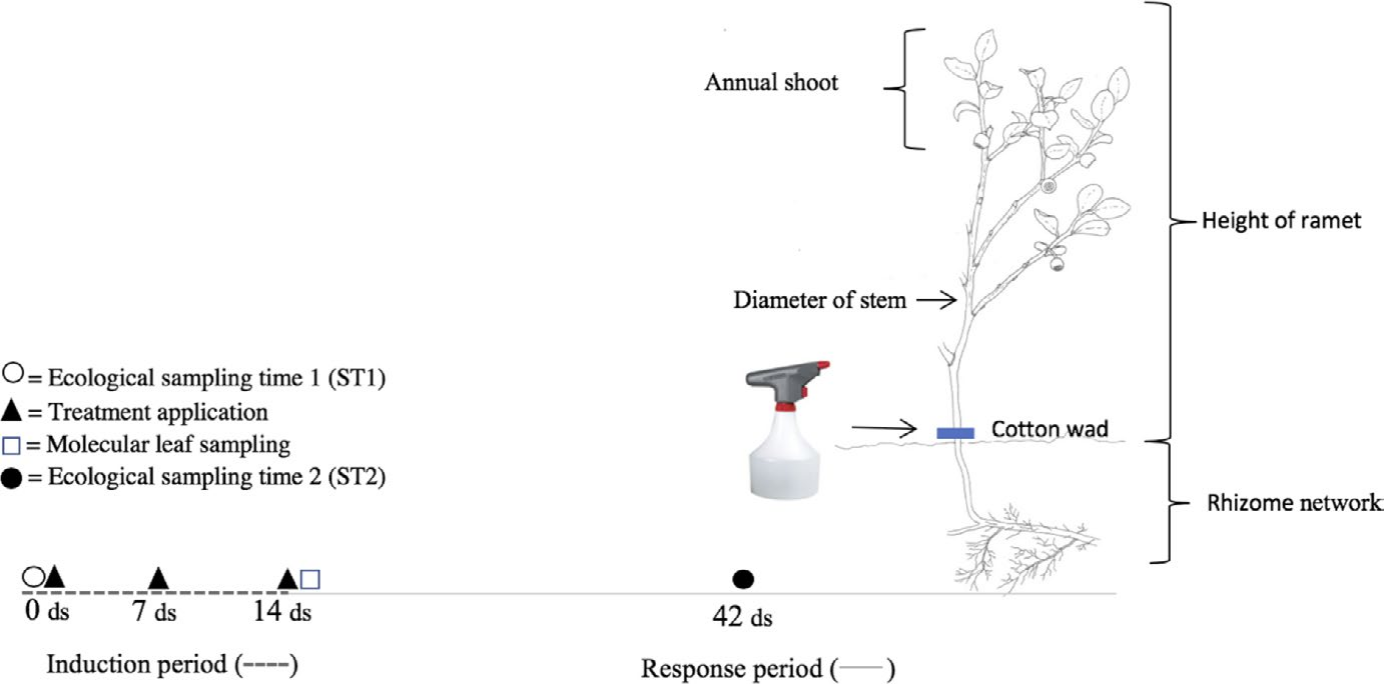
A bilberry ramet including size recordings, and timeline for the induction and response period when the measurements were recorded. ds: days

For the ecological analyses, the sampling procedure followed a similar methodology as in a previous ecological study (Benevenuto et al., 2018). Before the start of treatments in May/June 2016, sampling time 1 (ST1), all the experimental ramets were measured in terms of height from the ground to crown with a ruler and stem diameter at the surface using a digital caliper (Figure 2). We also counted the number of annual shoots, flowers, leaves, browsed shoots (plant shoots removed), and insect chewed leaves (leaves with insect chewing marks). We recorded the same variables 6 weeks (42 days) later, at sampling time 2 (ST2). At this subsequent recording, we also counted the number of berries. The same sampling procedure was repeated in 2017. Plant height (H), stem diameter (DS), and number of annual shoots (AS) were used to calculate dry mass (DM) of each ramet as a nondestructive estimate of plant size using the formula described by Hegland et al. (2010): log_2_(DM) = 1.41700 × log_2_(DS) + 0.97104 × log_2_(H) + 0.44153 × log_2_(AS + 1) – 7.52070.

To analyze gene expression, we collected 10 leaves from the apical part of each treatment in each block at 10:00 the day after the last treatment application, and they were immediately frozen in liquid nitrogen and stored at −80°C to avoid degradation. In June of 2017, a year after the last of the three treatment applications, leaf samples were collected in the same manner from all experimental plants. Samples were subsequently transferred to RNAlater‐ICE (Life Technologies, Carlsbad, CA) and moved to −20°C prior to RNA isolation according to the manufacturer's protocol, as detailed below.

### 2.4 Gene expression analysis

Collected samples were randomly separated into three biological groups, for each elevational site (Low; Medium; and High) and year (2016 and 2017) of study. Each biological group was composed by a pool of five different plants, a total of 15 plants/site/year. Total RNA from each group was extracted using the RNeasy Plant Mini Kit (Qiagen, Valencia, CA, USA) according to the manufacturer's directions. The total RNA was eluted in 100 µl sterile dH_2_O and quantified using a NanoDrop ND‐1000 spectrophotometer (NanoDrop Products; Wilmington, DE, USA). The cDNA was synthesized using 100 ng of total RNA per reaction and the Superscript VILO cDNA synthesis kit (Invitrogen, Carlsbad, CA, USA) according to the manufacturer's protocol.

A list of target genes for real‐time PCR and the primers used in the analysis are provided in Table S1. The selection of these eight genes involved in defense and growth‐related pathways was based on our previous RNA‐seq study of MeJA‐treated wild bilberry plants (Benevenuto et al., 2019). Defense target genes, involved in circadian rhythm, phenylpropanoid, tyrosine, flavonoid, and anthocyanin biosynthesis pathways, are shikimate O‐hydroxycinnamoyltransferase (*SHIKIMATE*), tyrosine aminotransferase (*TYR*), leucoanthocyanidin dioxygenase (*LDOX*), UDP‐glycosyltransferase (*UDP*), MYB‐related transcription factor LHY (*LHY*), and flavonoid 3′,5′‐hydroxylase (*FLAV*). Growth‐related target genes involved in photosynthesis and nitrogen metabolism are photosystem II PsbW (*PHOTO*) and glutamine synthetase chloroplastic (*GLU*).

Several genes were tested to be used for normalization, based on those commonly used in publications and our own transcriptome data (i.e., those not differentially expressed). These were β‐tubulin (*TUB*), metallothionein (*MET*), an F‐box gene (*Fbox*), ubiquitin‐conjugating enzyme E2 28 (*Ubc28*), and glyceraldehyde‐3‐phosphate dehydrogenase (*GAP*). The software package NormFinder was used to select the two most stable genes (*FBOX* and *GAP*) across our samples that were used for normalization. Ubc28 was also found to be stable and was used as the interplate calibrator. The gene sequences used for primer design were selected from the RNA‐seq de novo assembly of the bilberry transcriptome (Benevenuto et al., 2019), because the complete genome sequence of this species is not available. The primers were designed based on the predicted coding sequence of each target gene by using the online tool PrimerQuest (Integrated DNA Technologies Inc., Skokie, IL, USA). Real‐time PCRs were set up using the Power SYBR green PCR master mix (Applied Biosystems, Foster City, CA, USA) according to the manufacturer's directions and run on a QuantStudio 5 Real‐Time PCR System (Applied Biosystems). Thermocycling conditions were 50°C for 2 min; 95°C for 10 min; and 40 cycles at 95°C for 15 s, 60°C for 1 min, with melt curve set at 95°C for 15 s, 60°C for 1 min, 95°C for 30 s, and 60°C for 15 s. There were three biological replicates (pool of five plants) of each sample; in addition, three technical replicates were run for each biological replicate. Relative expression levels were calculated, based on the average cycle threshold (Ct) of the technical replicates for each biological replicate, by the ΔΔCt method using the QuantStudio Design & Analysis Software v1.4.3 (Applied Biosystems).

### 2.5 Data analyses

For the ecological data, the response variables of interest represent the change in herbivory and growth measures between the two sampling occasions of each year. At each sampling occasion, we measured the proportion of chewed leaves (number of chewed leaves/total number of leaves), proportion of browsed shoots (number of browsed shoots/total number of shoots), and biomass (through dry mass calculation), and then subtracted the values of ST1 (beginning of the season; Figure 2) from those of ST2 (end of the season; Figure 2) to obtain seasonal changes for each year. For these change variables, we used the *lme4* (Bates, Maechler, Bolker, & Walker, 2014) and *mixlm* (Liland & Sæbø, 2014) libraries in R (R Core Team, 2016) to fit generalized linear mixed effect models with Gaussian error distribution and identity link and to perform posterior ANOVA, respectively. Although a binomial error distribution is often more appropriate for proportion data, our *change in proportion* variables produced values beyond the usual 0 and 1 boundaries, and a Gaussian distribution was considered more appropriate. The treatment effects on reproduction were analyzed yearly, based on records from the last census in each year and subsequent calculation of fruit set (proportion of berries to flowers). Models on fruit set were fitted to generalized linear mixed models with binomial error distribution, since proportional values were bound by 0 and 1. For all models, we entered treatment (MeJA vs. control), site (Low, Medium, and High), and Year (2016 and 2017), with all interaction terms, as fixed effects. Block within each elevational site was fitted as a random effect in the models. To account for the possibility that plant size variation affected the plant response to treatments, covariates were also included in the models: total number of leaves at ST1 (for insect herbivory model), total number of shoots at ST1 (for mammalian herbivory model), total biomass at ST1 (for the growth model), and total number of flowers at ST2 (for the reproduction model). A visual inspection of residual plots did not reveal any obvious deviation from homoscedasticity or normality for any models. To determine the presence of an interaction effect, we performed likelihood ratio tests of the full model against the model without the effect and/or interaction in question. Considering the significant interaction terms in all models, we assessed the statistical significance (p‐values) of single factors and its respective interactions by using the ANOVA tables for each model (Table S2). In order to easily visualize MeJA treatment effects, seasonal effect sizes representing the mean difference between control and MeJA‐treated plants (control minus MeJA) for each response variable in each site and year are also presented. When the effect size was significant (ANOVA *p* < .05) for more than one site, differences and mean separations between elevational sites within each year were analyzed by Tukey *post hoc* comparisons (*p* < .05).

We also analyzed the effect of elevational gradient on the mean values of all plant (MeJA and control) response variables taken at ST2 across the two years to find effects that we could not see in models of seasonal changes, such as herbivory pressure/intensity, plant size, and total number of flowers and berries across the elevational gradient. The exception to this was total flowers, which we calculated as the mean of the total number of flowers over the two sampling sessions. That is because at ST2 most plants were in the fruit development stage, not presenting flowers at this time. Linear mixed models were fitted for each of these response variables by using treatment and site as fixed predictor variables, and block within each elevational site as random effect. After models were fitted, they were subjected to ANOVA followed by posterior mean separation by Tukey *post hoc* comparisons (*p* < .05).

For gene expression analyses, normalized relative expression values were log‐transformed to satisfy the assumptions of linear modeling (homogeneity of variance and normality in the residual distributions). Linear models were fitted with the *lme4* package (Bates et al., 2014) in R (R Core Team, 2016) to each target gene. Log‐transformed relative expression levels of each gene were used as the response variable, while treatment (MeJA or control), site (Low, Medium, and High), and Year (2016 and 2017) were the explanatory variables. A factorial ANOVA was conducted with the *mixlm* package (Liland & Sæbø, 2014) to determine whether there was an interaction effect between the three predictor variables (factors) (Table S2). Differences and means within each target gene were analyzed by Tukey *post hoc* comparisons (*p* < .05).

## 3 RESULTS

### 3.1 Ecological responses

**Figure 3:**
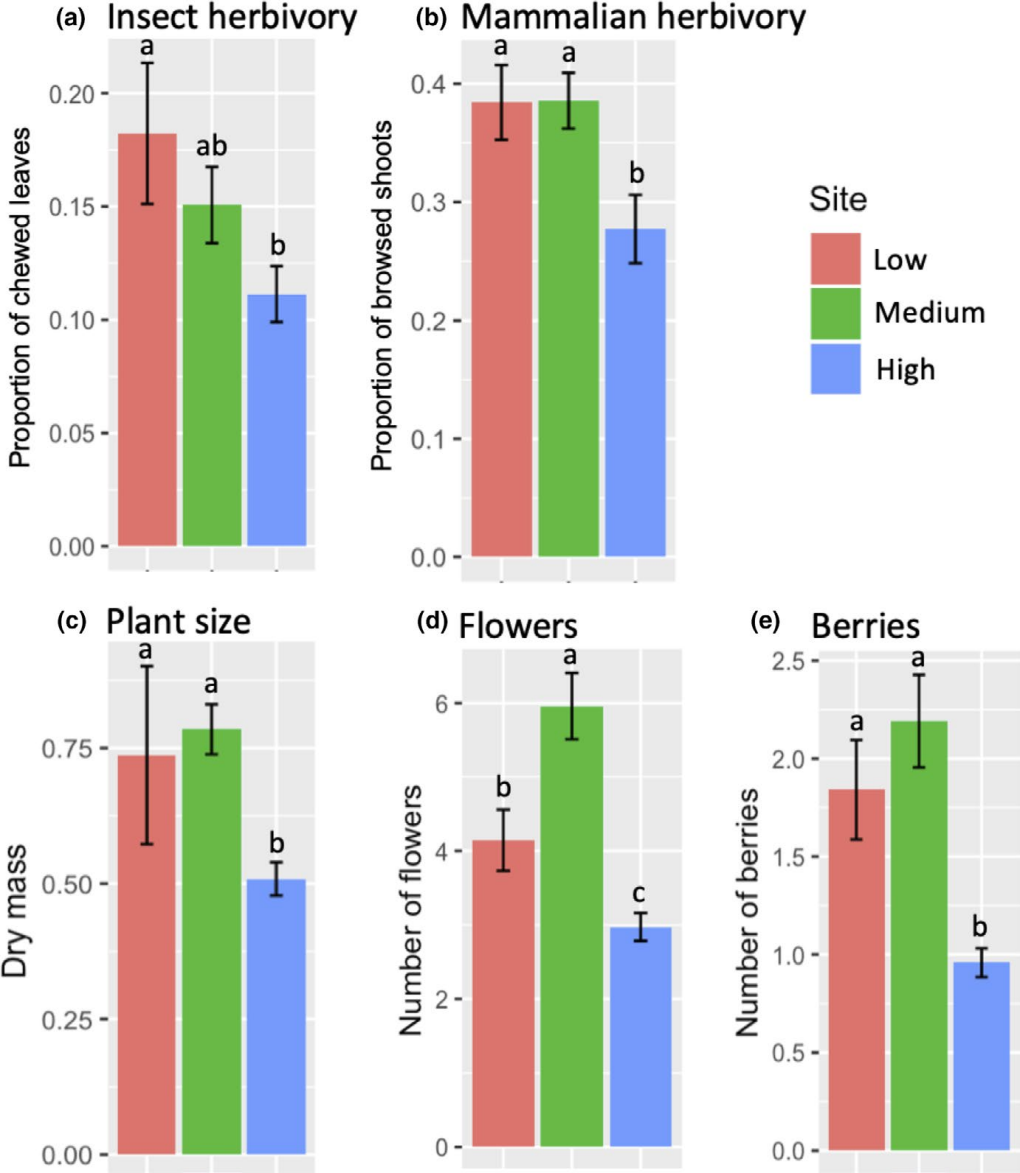
Mean of total herbivory, plant size, number of flowers, and berries across an elevation gradient over two years (2016 and 2017). Means of each response variable were calculated from the values collected from all plants (MeJA and control) in the end of the season (Aug‐Sep; sampling time 2) in both years of study. Error bars represent standard error of the mean. Different letters represent mean separation between sites (Tukey *p* < .05)

Total insect herbivory at the Low site (0.18 ± 0.03 [*SE*] proportion of chewed leaves) was 60% higher than at the High site (0.11 ± 0.01) but not significantly different from the Medium site (0.15 ± 0.02) (Figure 3a). Mammalian herbivory was 40% greater at the Low (0.39 ± 0.03 proportion of browsed shoots) and Medium (0.39 ± 0.02) sites than the High site (0.27 ± 0.03) (Figure 3b). Plant size was ~ 50% larger in Low‐elevation (0.74 ± 0.14 dry mass log_2_) and Medium‐elevation (0.78 ± 0.04) than at the High‐elevation sites (0.50 ± 0.02) (Figure 3c). Bilberry plants from the Medium site produced 6 ± 0.4 flowers, which is 40% more than plants from the Low site (4.1 ± 0.3) and twice as much as plants at the High site (3 ± 0.1) (Figure 3d). Bilberry plants at the Low (1.9 ± 0.5 berries) and Medium (2.4 ± 0.5) sites produced about twice as many berries compared with plants from the High site (0.9 ± 0.2) (Figure 3e).

**Figure 4:**
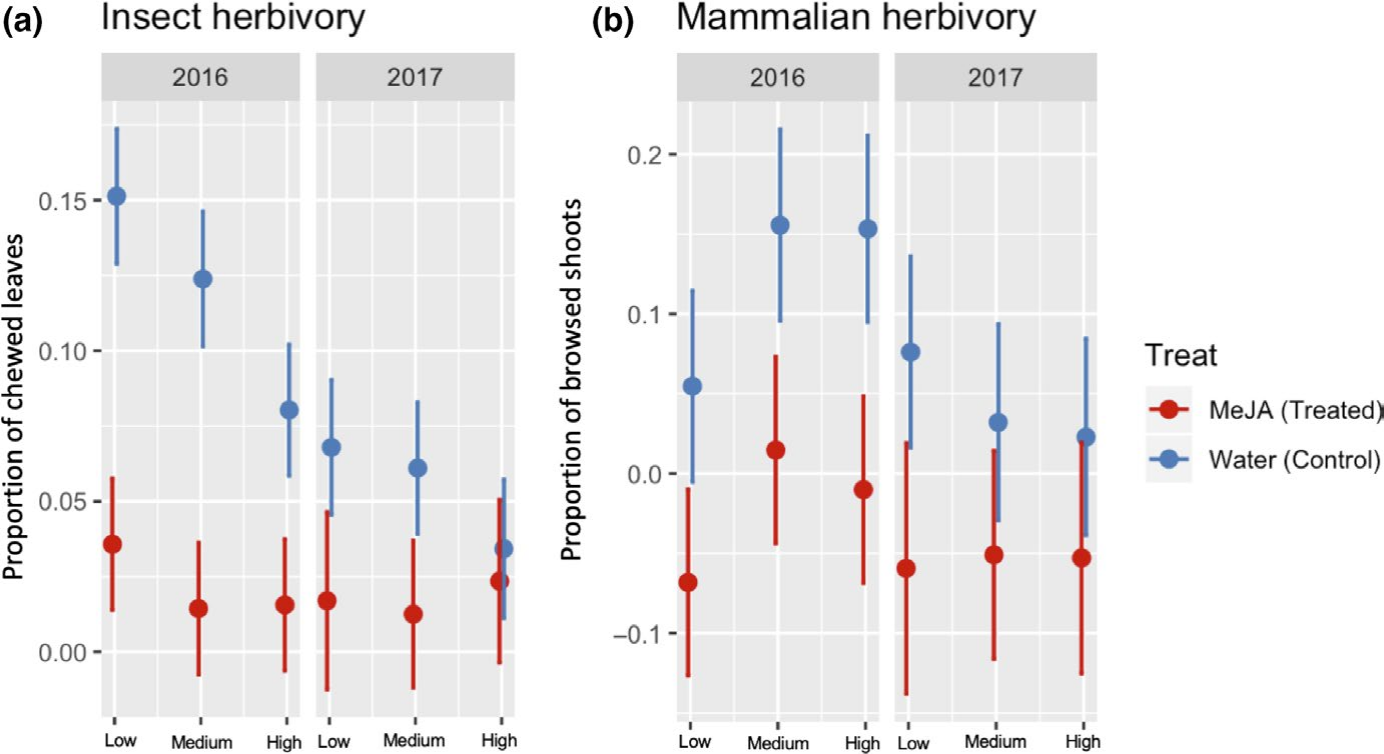
Seasonal changes in insect (a) and mammalian (b) herbivory of wild bilberry plants (*Vaccinium myrtillus* L.) in response to MeJA treatment across a elevational gradient (Low, Medium, and High) for two consecutive years (2016 and 2017). Seasonal changes in proportion of insect chewed leaves (no. of chewed leaves/total number of leaves) and proportion of deer browsed shoots (no. of browsed shoots/total number of shoots) were calculated as difference between value at the end of the season (Aug‐Sep; sampling time 2) minus value at the beginning of the season (May‐June; sampling time 1) in each year. Error bars represent standard error of the mean

**Figure 5:**
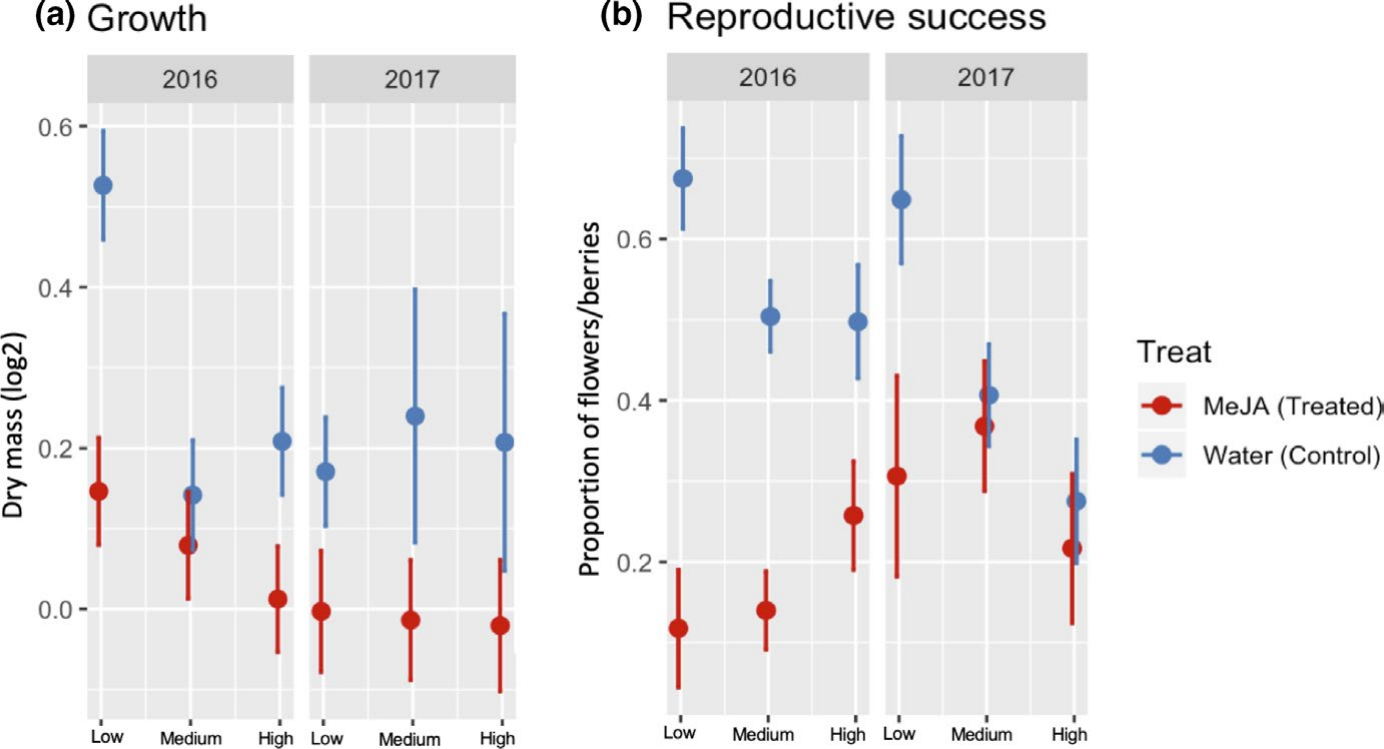
Seasonal changes in growth (a) and reproduction (b) of wild bilberry plants (*Vaccinium myrtillus* L.) in response to MeJA treatment across a elevational gradient (Low, Medium, and High) for two consecutive years (2016 and 2017). Seasonal growth in dry mass was calculated as difference between value at the end of the season (Aug‐Sep; sampling time 2) minus value at the beginning of the season (May‐June; sampling time 1) in each year. Reproduction is the proportion of total number of berries/total number of flowers in each year. Error bars represent standard error of the mean

Seasonal changes in the proportion of chewed leaves (insect herbivory) (Treat*site*Year effect: *F* = 12.09; *p* = .01), proportion of browsed shoots (mammalian herbivory; Treat*site*Year effect: *F* = 10.58; *p* = .03), dry mass (growth; Treat*site*Year effect: *F* = 14.19; *p* = .006), and fruit set (Treat*site*Year effect: *F* = 18.59; *p* = .0009) showed significant interaction effects among all three factors involved (Table S2). In the year of treatment (2016), MeJA‐treated plants had significantly lower proportions of insect and mammalian herbivory than control plants across all elevational sites, although to varying degrees. The difference was ~80% higher for plants from Low and Medium sites than plants growing at the High site (Table 1; Figure 4a). Differences found in mammalian herbivory did not significantly differ between elevational sites (Table 1; Figure 4b). MeJA‐treated plants from Low and High sites grew significantly less than the control in 2016, whereas no significant effect was found at the Medium site. Effect size of growth was ~80% greater at the Low site than the High site (Table 1; Figure 5a). Fruit set in 2016 was reduced by the MeJA treatment in all sites, although the effect size was significantly stronger at the Low site, followed by the Medium and the High sites (Table 1; Figure 5b).

**Table 1:** Effect sizes of seasonal changes in herbivory, growth, and reproduction of wild bilberry plants (*Vaccinium myrtillus* L.) across the season in response to MeJA treatment and elevational gradient for two consecutive years

Effect sizes (control minus MeJA)	2016			2017		
	L	M	H	L	M	H
Insect chewed leaves (proportion)	0.11 ± 0.02 a	0.11 ± 0.02 a	0.06 ± 0.01 b	0.05 ± 0.01 a	0.05 ± 0.02 a	NS
Deer browsed shoots (proportion)	0.13 ± 0.03 a	0.15 ± 0.04 a	0.16 ± 0.04 a	0.12 ± 0.04	NS	NS
Growth (log_2_ g dry mass)	0.37 ± 0.04 a	NS	0.2 ± 0.04 b	0.17 ± 0.05 a	0.25 ± 0.07 a	0.2 ± 0.07 a
Reproduction (fruit set)	0.6 ± 0.06 a	0.4 ± 0.03 b	0.21 ± 0.05 c	0.34 ± 0.05	NS	NS

Note

Changes in proportion of insect chewed leaves (no. of chewed leaves/total number of leaves); proportion of deer browsed shoots (no. of browsed shoots/total number of shoots), and growth (dry mass) were calculated as difference between value at the end of the season (Aug–Sep) minus value at the beginning of the season (May‐June) each year. Reproduction (fruit set) was calculated as the proportion between total number of berries/total number of flowers in each year. Effect sizes represent the mean difference (with standard error) between control and MeJA seasonal effect for each response variable within each site and year. “NS” indicates no significant difference (ANOVA *p* < .05) in effect size for the specific comparison within each site and year. For significant effect sizes, different letters represent mean separation between elevational sites within each year (Tukey *p* < .05).

One year after treatment (2017), insect herbivory remained lower in MeJA‐treated plants than the control at Low and Medium sites. Effect sizes were not significantly different between sites (Table 1; Figure 4a). Mammalian herbivory was also significantly lower in 2017 for MeJA‐treated plants, but only for those located at the Low site (Table 1; Figure 4b). Negative effects of MeJA treatment on dry mass were maintained for plants growing at the Low and High sites in 2017; however, in contrast to 2016, MeJA‐treated plants from the Medium site also exhibited lower growth rates than the control plants in 2017. Effect sizes of seasonal growth did not differ between sites one year after treatment (Table 1; Figure 5a). MeJA‐treated plants maintained a significant reduction in fruit set at the Low site in 2017 (Table 1; Figure 5b).

### 3.2 Gene expression

Considering single‐factor effects (without interaction), all defense‐related genes tested in our study were upregulated in response to the MeJA treatment, while photosynthesis‐ and nitrogen metabolism‐related genes were downregulated (Table S3; treatment effect). The single effect for “site” occurred because all bilberry plants located at the High site had significantly higher levels of expression for *FLAV*, *LHY*, *SHIKIMATE*, and *LDOX* than plants from the Low site (Table S3; site effect). Regarding “Year” as a factor, all target genes tested were more highly expressed in the year of treatment (2016) than a year later (2017) (Table S3; Year effect).

**Figure 6:**
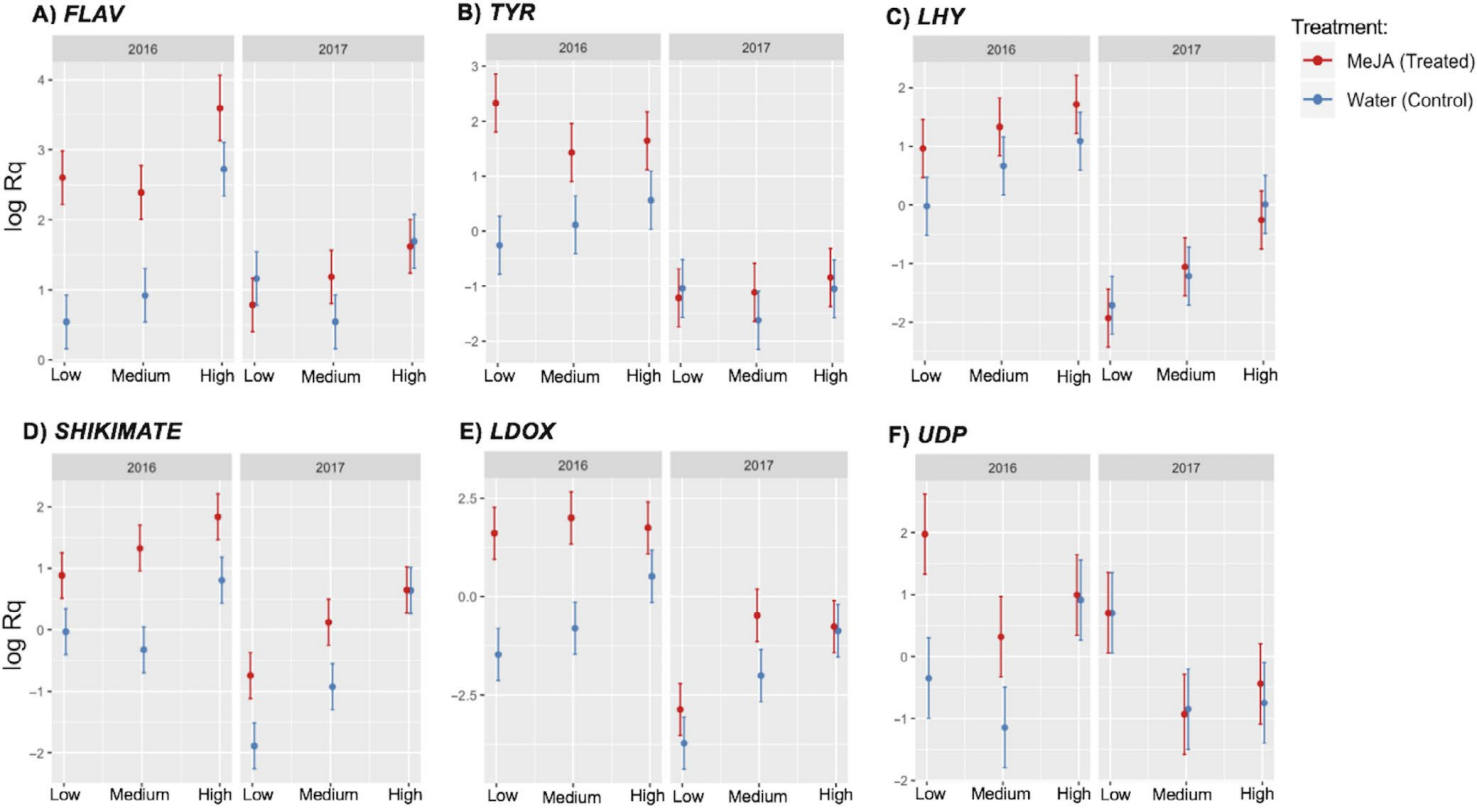
Relative expression levels of six (a–f) defense‐related target genes in bilberry plants (*Vaccinium myrtillus* L.) in response to MeJA treatment and elevational gradient (Low, Medium, and High) for two consecutive years (2016 and 2017). The log of relative expression shown is the mean for each treatment in each elevational site and in each year; error bars represent confidence interval at 95%. Full names of target genes are presented in Table S1

**Figure 7:**
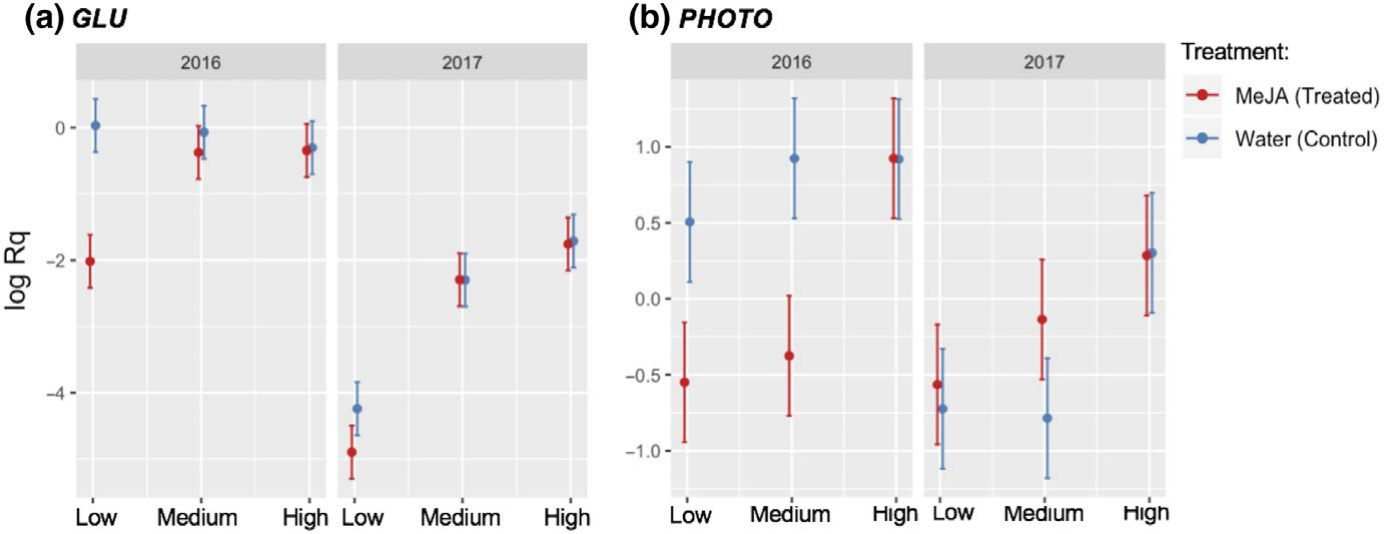
Relative expression level of two (a–b) growth‐related target genes in bilberry plants (*Vaccinium myrtillus* L.) in response to MeJA treatment and elevational gradient (Low, Medium, and High) for two consecutive years (2016 and 2017). The log of relative expression shown is the mean for each treatment in each elevational site and in each year; error bars represent confidence interval at 95%. Full names of target genes are presented in Table S1

All defense‐related target genes showed a significant (*p* < .05) or strong trend (*p* < .1) interaction effect between the factors involved in our study, which means that their expression level depends on the interaction between specific treatment, elevational site, and time after induction. *FLAV* (Treat*site*Year effect: *F* = 5.81; *p* = .009), *TYR* (Treat*site*Year effect: *F* = 4.66; *p* = .02), *LHY* (Treat*Year effect: *F* = 4.98; *p* = .03), *SHIKIMATE* (Treat*site*Year effect: *F* = 3.11; *p* = .06), and *LDOX* (Treat*Year effect: *F* = 17.33; *p* = .0003; and Treat*site effect: *F* = 6.43; *p* = .005) were similarly affected: MeJA‐treated plants showed higher expression levels than control plants at all sites to varying degrees and mainly for 2016 (Table S3; Table 2; Figure 6a–e). In the same year, the MeJA treatment also induced the upregulation of *UDP* (Treat*site*Year effect: *F* = 4.33; *p* = .02) in bilberry plants located only at the Low and Medium sites (Table 2; Figure 6f). *GLU* (Treat*site*Year effect: *F* = 3.57; *p* = .04) and *PHOTO* (Treat*site*Year effect: *F* = 6.79; *p* = .004) expression levels also depended on the interaction between the three factors; in 2016, MeJA treatment downregulated the expression of *GLU* only in plants at the Low site (Table 2; Figure 7a). MeJA‐treated plants from Low and Medium sites showed reduced expression levels of *PHOTO* compared with the controls at each site (Table 2; Figure 7b).

**Table 2:** Differentially expressed genes (log_2_ fold change) in wild bilberry plants (*Vaccinium myrtillus* L.) in response to MeJA treatment and elevational gradient for two consecutive years

	Log_2_ fold change of gene expression (log2FC)					
	2016			2017		
	Low	Medium	High	Low	Medium	High
Defense‐related genes						
*FLAV*	2.84	2.00	1.18	NS	0.91	NS
*TYR*	3.75	1.86	1.5	NS	0.72	NS
*LHY*	1.49	0.92	0.89	NS	NS	NS
*SHIKIMATE*	1.28	2.33	1.48	0.94	1.6	NS
*LDOX*	3.74	4.06	1.87	1.32	2.27	NS
*UDP*	3.36	2.00	NS	NS	NS	NS
Growth‐related genes						
*GLU*	−2.83	NS	NS	−1.12	NS	NS
*PHOTO*	−1.51	−1.88	NS	NS	0.85	NS

Note

A positive value indicates that the respective gene was significantly upregulated for the MeJA‐treated plant. A negative value means that the respective gene was significantly downregulated for the MeJA‐treated plant. “ns” indicates that no significant difference (ANOVA *p* < .05) between MeJA‐treated and control plant was found for the relative expression of the respective gene on that specific comparison.

One year after induction (2017), upregulation of *FLAV*, *TYR*, *SHIKIMATE*, and *LDOX* defense genes was maintained for the MeJA‐treated plants in Low and/or Medium sites. In the High site, no significant changes were found for any defense genes tested (Table 2; Figure 6a–e). *GLU* maintained the same response from 2016, with reduced expression in MeJA‐treated plants at the Low site (Table 2; Figure 7a). MeJA‐treated plants at the Medium site had significantly higher levels of *PHOTO* expression than control plants one year after treatment (2017) (Table 2; Figure 7b).

## 4 DISCUSSION

The plant defense system in bilberry varied over an elevational gradient. Induced defenses were strongly activated for plants growing at the optimum (Medium site) and suboptimal lower elevation (Low site), suggesting that these plants have more resources available to invest in defense against herbivore attack, compared with high‐elevation plants growing under colder and resource‐limiting conditions. The MeJA activation of induced defenses was strongest at the Low site, and this was supported by the molecular mechanisms found: Low‐elevation plants effectively reduced seasonal growth, fruit set, and herbivory rates, while significantly downregulated growth‐related genes and upregulated defense‐related genes in response to MeJA treatment. These combined responses strongly suggest resource allocation from growth and reproduction to induced defenses (trade‐off at ecological and molecular levels), especially for plants growing at the warmest low‐elevation site (*Prediction I*). Moreover, we showed that these effects persisted for at least one year after treatment. On the other hand, bilberry plants growing at the suboptimal and coldest upper elevation (High site) invested more in constitutive than induced defenses, indicating that they are under constant “alert.” This was supported by the small effect sizes of changes in growth, fruit set, and herbivory resistance, and the consistently high expression levels of defense‐related genes in both MeJA‐treated and untreated plants at high elevation (*Prediction II*).

### 4.1 Herbivory and defense

Ecological responses related to defense showed that, in the year of treatment (2016), plants growing under optimal (Medium site) and suboptimal lower elevations (Low site) had the largest effect sizes (differences between control and MeJA) in seasonal changes of herbivory. Although MeJA treatment also appeared to increase herbivore resistance in bilberry plants from the suboptimal upper elevation (High site) in the year of treatment, this effect was considerably lower when compared to plants from lower elevations (Low and Medium sites). A year after treatment (2017), significant herbivore resistance effects persisted in MeJA‐treated plants from Low and Medium sites, but the same was not found at the highest elevation. This finding corroborates recent studies showing that induced defenses decline with increasing elevation (Moreira et al., 2014, 2018; Pellissier et al., 2016), and partially supports the optimal defense hypothesis such that allocation of resources to inducible defenses, but not to constitutive defenses, in bilberry seems to depend on the greater risk of herbivory at lower elevations. This suggests that herbivory at lower elevations might not be predictable.

We found molecular responses at gene expression level that could help explain the ecological findings. Expression of important defense‐related genes was upregulated by MeJA (positive log2FC) across all sites in 2016. This was particularly evident in the genes involved in the phenylpropanoid, flavonoid, and anthocyanin biosynthesis pathways (i.e., *FLAV*, *TYR*, SHIKIMATE, and *LDOX*). Bilberry plants are particularly known for a naturally high content of anthocyanins, pigments present in fruits, leaves, and flowers, which are considered as important component in defenses against herbivore attack (Lattanzio, Lattanzio, & Cardinali, 2006). The *SHIKIMATE* gene acts early in the phenylpropanoid biosynthesis pathway, forming important precursors of different classes of secondary metabolites, including anthocyanins (Hoffmann et al., 2004). *FLAV* and *LDOX* act later in the flavonoid biosynthesis pathways, encoding enzymes to catalyze precursors for the synthesis of flavones and flavonols. Besides secondary metabolites, MeJA also induced *LHY,* an important morning‐specific transcription factor involved in flowering time, in 2016, especially in low‐elevation bilberry plants. Changes in expression of LHY‐related transcription factors cause arrhythmicity in the expression of other clock‐regulated genes, which can alter leaf movement and flowering aiming to avoid insect herbivory of flower tissues (Alabadí, Yanovsky, Más, Harmer, & Kay, 2002; Green & Tobin, 1999; Mizoguchi et al., 2002).

Our results indicate that bilberry plants rapidly invest in the synthesis of these metabolites, in response to herbivory (as simulated by MeJA induction), especially at the optimal medium and warmer low‐elevation site. Although significant effects of defense induction at gene level were found along all elevational gradients, as shown in the ecological results, plants growing at the low and medium elevations showed stronger upregulation of defense genes (higher log2FC; Table 1) than high‐elevation plants. In 2017, upregulation of most defense‐related genes persisted in MeJA‐treated plants from the low and medium elevations, while no differences were found in plants located in the highest elevation. Thus, such molecular results together with ecological findings of reduced herbivory at the optimal medium and warmer low‐elevation sites can partly be interpreted as allocation of resources in response to reduced environmental pressure.

### 4.2 Growth and reproduction

Ecological results related to growth and reproduction showed that, in the year of treatment (2016) and one year later (2017), bilberry plants growing at the suboptimal lower elevation (Low site) presented the largest effect sizes in biomass and fruit set measurements, compared to plants located at the medium and high elevations. As found in measurements related to defense, even though MeJA treatment affected seasonal growth and reproduction in plants growing across all elevational gradients, evidence of resource allocation to defense was less apparent in MeJA‐treated plants from Medium and High sites than for plants at the lower elevation.

As for defense, our molecular findings for genes related to growth and development support ecological responses in relation to our first prediction. GLU had its expression significantly downregulated in MeJA‐treated plants growing at the low elevation in the year of treatment (2016) and one year later (2017). GLU plays a crucial role in nitrogen metabolism via assimilation of ammonium obtained from photorespiration (Bernard & Habash, 2009). Most of the assimilated nitrogen in plants is invested in photosynthesis; hence, nitrogen assimilation and photosynthetic capacity are strongly correlated (Makino, Nakano, Mae, Shimada, & Yamamoto, 2000; Makino, Sakuma, Sudo, & Mae, 2003; Nunes‐Nesi, Fernie, & Stitt, 2010). MeJA‐treated plants growing at the Low site rapidly reduced the expression of GLU, probably to allocate nitrogen‐related resources from growth and development to storage in case of herbivore attack as part of induced defenses. PHOTO, an important gene from photosystem II, was also downregulated by MeJA in bilberry plants from Low and Medium sites in the year of treatment. However, the same effect did not persist until the next season. The downregulation of PHOTO gene, as well as substantial decreases in seasonal growth found in our study, suggests that the inhibitory effect of MeJA on photosynthesis is effective due to the reduction in the light‐harvesting complexes, consequently decreasing carbon fixation. Our findings corroborate with previous transcriptomic studies, where PHOTO and other important chlorophyll‐related genes from the photosystem II complex were downregulated in response to MeJA treatment in wild bilberries (Benevenuto et al., 2019; Bilgin et al., 2010). At the very last, high‐elevation genotypes showed to not immediately downregulate growth‐related genes in response to MeJA treatment as we have seen for low‐elevation genotypes. This could be understood as another indication that such plants do not rapidly invest in induced defenses as bilberries from lower elevations. Although a little complex, such gene expression results underpin molecular mechanisms underlying ecological responses found regarding the type of defense strategy expressed in bilberry plants across an elevational gradient in the boreal ecosystem.

### 4.3 Trade‐offs along the elevational gradient

Our findings indicate an effective trade‐off between growth/reproduction and defense in bilberry plants under herbivory pressure from warmer low‐elevation environments in the year of treatment, suggesting an investment strategy of protecting against future or persistent herbivore attacks. In a previous study exploring the transcriptional profiling of MeJA‐induced defense responses in bilberry, we found evidences for “gene regulation trade‐off”: the upregulation of genes involved in important defense‐related pathways and the corresponding downregulation of genes related to growth and nitrogen metabolism pathways (Benevenuto et al., 2019). Trade‐offs persisted one year after MeJA induction only at the lowest elevation site. In 2017, downregulation of *GLU* correlated with the upregulation of *SHIKIMATE* and *LDOX* defense genes for plants located at the Low site, indicating that gene regulation trade‐offs between defense and growth‐related genes can be multiannual in warmer suboptimal conditions. Ecological responses also showed trade‐offs between growth/reproduction and herbivory resistance one year after induction in low‐elevation bilberry plants of the boreal system. In general, the effects of MeJA‐induced defenses disappeared in plants growing at the colder and resource‐limiting high elevation one year after treatment.

Previous studies have shown that high temperature can directly affect the synthesis of secondary metabolites involved in defense (Estiarte et al., 1999; Gouinguené & Turlings, 2002; Guo et al., 2012; Mosolov & Valueva, 2011; Sun et al., 2011; Veteli et al., 2002). Indeed, our findings suggest that: when induced under variable environmental conditions, bilberry plants in the boreal system appear to invest in the upregulation of defense‐related pathways (i.e., synthesis of secondary metabolites), while downregulating growth‐related genes, and consequently reducing seasonal growth and reproduction to increase herbivory resistance. However, although still significant in low‐elevation plants, these effects seem to be relaxed one year after induction. Induced defense responses can persist from hours and days to years, depending on the plant species, previous herbivory pressure, and life history of the plant (Haukioja, Suomela, & Neuvonen, 1985; Karban & Baldwin, 1997). Besides, as abiotic factors can profoundly influence plant development through changes in metabolic rates (O'Connor, 2009), and phenotypic plasticity is a developmental phenomenon, spatial or temporal variation in temperature can also affect the speed, magnitude, and costs (allocation of resources from growth/reproduction to defense) associated with induced defenses (Trussell, David, & Smith, 2000). Our results suggest that bilberry plants are more responsive to induced defenses in the suboptimal lower elevation, where the average temperature is higher and the timing of snow melt is earlier, by allocating resources from growth and reproduction to effective herbivory defense for at least one year after induction. This result corroborates with our previous ecological study showing that the induced defense system in low‐land bilberry plants functions in a multiannual manner (Benevenuto et al., 2018).

As predicted by the resource availability hypothesis, high‐elevation bilberry plants invested more in constitutive than induced defenses. Small effect sizes for some of the ecological response variables tested (i.e., insect herbivory and fruit set) indicate that high‐elevation plants may invest limited resources to defense at all times. At the molecular level, all bilberry plants (MeJA‐treated and control) growing at the high‐elevation showed high basal levels of expression for most defense genes tested (i.e., *FLAV*, *LHY*, *SHIKIMATE*, and *LDOX*). Furthermore, we observed that general herbivory pressure was less intense (i.e., lower total proportion of chewed leaves) at the high‐elevation than the low‐elevation site of the elevational gradient, consistent with recent studies (Pellissier et al., 2012; Rasmann, Pellissier, Defossez, Jactel, & Kunstler, 2014). This suggests that bilberry plants growing in suboptimal upper elevations are under a constant state of “alert” (constitutive defenses), possibly because of the resource‐limiting and stressful environmental conditions (i.e., low nutrients, cool average temperatures, late timing of snow melt, and consequent short‐growing season); as well as lower herbivory pressure in subalpine habitats of the boreal system (Moreira et al., 2018), contrary to optimal defense theory assumptions (Rhoades, 1979). The combination of these environmental conditions, biotic and variable abiotic factors, likely affects the “decisions” regarding the type of defense strategy expressed in bilberry plants from a reliance on constitutive defenses instead of inducible defenses (Galmán et al., 2018). Plant species adapted to stressful environments are predicted to increase allocation to expensive constitutive defenses relative to induced defenses, as the energetic demands to replace tissues consumed by herbivores are much higher under such limiting resource environment (Moreira et al., 2014; Pellissier et al., 2016). Recent studies have shown that defense trade‐offs are complex and can be interpreted as result of regulatory “decisions” by plants aiming to fine‐tune their phenotype in response to multiple environmental conditions (Galmán et al., 2018; Züst & Agrawal, 2017). Bilberry plants from higher elevations may have developed consistent levels of plant defenses as an adaptation process to severe climatic conditions, which indirectly confer increased resistance to herbivores. Therefore, high‐elevation plants may not be adapted, but ecologically fitted to future herbivore pressure due to possible rapid shifts in herbivore range under climate change (Rasmann, Pellissier, et al., 2014).

## 5 CONCLUSION

We showed that bilberry defense responses are modulated by the combination of climate and herbivory pressure at both the ecological and molecular levels in the boreal system. High‐elevation plants invested more in constitutive defenses, whereas low‐elevation plants relied strongly on induced defenses. Although herbivory pressure is lower at high elevations of the boreal system, the cooler average temperatures and limiting resource availability were associated with constantly “alert” bilberry plants, whether treated with MeJA or not. The plants located at low elevations, where herbivory rates and seasonal temperature are higher, appeared to be more responsive to defense induction by effectively investing resources away from growth and reproduction to induced antiherbivory defenses. This result suggests that under increasingly warmer conditions and higher herbivory pressure, bilberry plants may respond by altering their defense strategy.

Our results agree with previous studies showing that plant defensive traits are influenced by, and can adapt to, a combination of both biotic (i.e., herbivory pressure and biodiversity) and abiotic (i.e., temperature and resource availability) factors along an elevational gradient (Moreira et al., 2014; Pearse & Hipp, 2012). Under the current scenario of global warming (IPCC, 2018; Serreze et al., 2000), suboptimal upper elevation is likely to become the new optimal growing environment for most species in the boreal system. If so, the defense system of low‐ and medium‐elevation plants that migrate to higher elevations might encounter novel abiotic (e.g., harsher environment and less resource availability) and biotic (e.g., less diversity and herbivory pressure) conditions to which they need to adapt. On the other hand, rapid shifts of herbivore range to higher elevation due to increasing temperatures may result in limited damage to plants not adapted to such herbivory pressure. These combinations of events in response to climate change can consequently catalyze new ecological and coevolutionary dynamics through modulating plant–herbivore interactions in the subalpine zones of the boreal system. Plant–herbivore interactions are one of the major drivers of ecosystem functioning and diversity, and thus, the evolution of plant defense system has been suggested to sculpt such patterns (Ehrlich & Raven, 1964). The ecological and molecular work in this study has provided insights into current patterns of plant defense strategies along abiotic gradients. These approaches could be used to monitor migration and analyze changes in plant–herbivore relationships, which can help us to better predict possible modifications on the boreal ecosystem functioning during climate change.

## CONFLICT OF INTEREST

The authors declare that they have no competing interests.

## AUTHORS’ CONTRIBUTIONS

RFB led the bioinformatics and statistical analysis, interpretation of the data, and writing of the manuscript. All authors contributed to critical reading and editing of the manuscript. RFB, TS, SJH, MAG, SRM, and CR‐S conceived the initial idea, designed, and performed field work. RFB and JP led the RT‐qPCR portion of the project. All authors approved the final manuscript.

## Data Availability

All data generated for this study are publicly available at NCBI SRA under BioProject ID number PRJNA481170.

## References

[ece36074-bib-0001] Ahuja, I. , de Vos, R. C. H. , Bones, A. M. , & Hall, R. D. (2010). Plant molecular stress responses face climate change. Trends in Plant Science, 15(12), 664–674. 10.1016/j.tplants.2010.08.002 20846898

[ece36074-bib-0002] Alabadí, D. , Yanovsky, M. J. , Más, P. , Harmer, S. L. , & Kay, S. A. (2002). Critical role for CCA1 and LHY in maintaining circadian rhythmicity in Arabidopsis. Current Biology, 12(9), 757–761. 10.1016/S0960-9822(02)00815-1 12007421

[ece36074-bib-0003] Albert, T. , Raspé, O. , & Jacquemart, A.‐L. (2003). Clonal structure in *Vaccinium myrtillus* L. revealed by RAPD and AFLP markers. International Journal of Plant Sciences, 164(4), 649–655.

[ece36074-bib-0004] Albert, T. , Raspé, O. , & Jacquemart, A.‐L. (2004). Clonal diversity and genetic structure in *Vaccinium myrtillus* populations from different habitats. Belgian Journal of Botany, 164, 155–162.

[ece36074-bib-0005] Atlegrim, O. (1989). Exclusion of birds from bilberry stands: Impact on insect larval density and damage to the bilberry. Oecologia, 79(1), 136–139. 10.1007/BF00378251 28312824

[ece36074-bib-0006] Baldwin, I. T. (1999) Inducible nicotine production in native Nicotiana as an example of adaptive phenotypic plasticity. Journal of Chemical Ecology, 25(1):3–30.

[ece36074-bib-0007] Bates, D. , Maechler, M. , Bolker, B. , & Walker, S. (2014). lme4: Linear mixed‐effects models using Eigen and S4. R Package Version, 1(7), 1–23.

[ece36074-bib-0008] Benevenuto, R. F. , Hegland, S. J. , Töpper, J. P. , Rydgren, K. , Moe, S. R. , Rodriguez‐Saona, C. , & Seldal, T. (2018). Multiannual effects of induced plant defenses: Are defended plants good or bad neighbors? Ecology and Evolution, 8(17), 8940–8950. 10.1002/ece3.4365 30271557 PMC6157685

[ece36074-bib-0009] Benevenuto, R. F. , Seldal, T. , Hegland, S. J. , Rodriguez‐Saona, C. , Kawash, J. , & Polashock, J. (2019). Transcriptional profiling of methyl jasmonate‐induced defense responses in bilberry (*Vaccinium myrtillus* L.). BMC Plant Biology, 19(1), 70 10.1186/s12870-019-1650-0 30755189 PMC6373060

[ece36074-bib-0010] Bernard, S. M. , & Habash, D. Z. (2009). The importance of cytosolic glutamine synthetase in nitrogen assimilation and recycling. New Phytologist, 182(3), 608–620. 10.1111/j.1469-8137.2009.02823.x 19422547

[ece36074-bib-0011] Bidart‐Bouzat, M. G. , & Imeh‐Nathaniel, A. (2008). Global change effects on plant chemical defenses against insect herbivores. Journal of Integrative Plant Biology, 50(11), 1339–1354. 10.1111/j.1744-7909.2008.00751.x 19017122

[ece36074-bib-0012] Bilgin, D. D. , Zavala, J. A. , Zhu, J. , Clough, S. J. , Ort, D. R. , & Delucia, E. H. (2010). Biotic stress globally downregulates photosynthesis genes. Plant, Cell & Environment, 33(10), 1597–1613. 10.1111/j.1365-3040.2010.02167.x 20444224

[ece36074-bib-0013] Bokhari, S. A. , Wan, X.‐Y. , Yi‐Wei Yang, L. U. , Zhou, W.‐L. , & Liu, J.‐Y. (2007). Proteomic response of rice seedling leaves to elevated CO2 levels. Journal of Proteome Research, 6(12), 4624–4633. 17988085 10.1021/pr070524z

[ece36074-bib-0014] Chen, J. , Burke, J. J. , Velten, J. , & Xin, Z. (2006). FtsH11 protease plays a critical role in Arabidopsis thermotolerance. The Plant Journal, 48(1), 73–84. 10.1111/j.1365-313X.2006.02855.x 16972866

[ece36074-bib-0015] Cheng, C. , Gao, X. , Feng, B. , Sheen, J. , Shan, L. , & He, P. (2013). Plant immune response to pathogens differs with changing temperatures. Nature Communications, 4, 2530 10.1038/ncomms3530 PMC3901997 24067909

[ece36074-bib-0016] Coley, P. D. , Bryant, J. P. , Stuart, F. , & Chapin, F. S. (1985). Resource availability and plant antiherbivore defense. Science, 230(4728), 895–899. 10.1126/science.230.4728.895 17739203

[ece36074-bib-0017] Danell, K. , Bergstrom, R. , & Iedenius, L. (1994). Effects of large mammalian browsers on architecture, biomass, and nutrients of woody‐plants. Journal of Mammalogy, 75(4), 833–844. 10.2307/1382465

[ece36074-bib-0018] DeLucia, E. H. , Nabity, P. D. , Zavala, J. A. , & Berenbaum, M. R. (2012). Climate change: Resetting plant‐insect interactions. Plant Physiology, 160(4), 1677–1685. 10.1104/pp.112.204750 22972704 PMC3510101

[ece36074-bib-0019] Ehrlich, P. R. , & Raven, P. H. (1964). Butterflies and plants ‐ a study in coevolution. Evolution, 18(4), 586–608. 10.2307/2406212

[ece36074-bib-0020] Estiarte, M. , Penuelas, J. , Kimball, B. A. , Hendrix, D. L. , Pinter, P. J. , Wall, G. W. , … Hunsaker, D. J. (1999). Free‐air CO2 enrichment of wheat: Leaf flavonoid concentration throughout the growth cycle. Physiologia Plantarum, 105(3), 423–433. 10.1034/j.1399-3054.1999.105306.x

[ece36074-bib-0021] Flower‐Ellis, J. G. K. (1971). Age structure and dynamics in stands of bil‐berry (*Vaccinium**myrtillus* L.). PhD thesis. Royal College of Forest Ecology and Forest Soils, Research Notes, 9, 1–108.

[ece36074-bib-0022] Galmán, A. , Petry, W. K. , Abdala‐Roberts, L. , Butrón, A. , de la Fuente, M. , Francisco, M. , … Moreira, X. (2018). Inducibility of chemical defences in young oak trees is stronger in species with high elevational ranges. Tree Physiology, 39(4), 606–614. 10.1093/treephys/tpy139 30597091

[ece36074-bib-0023] Garibaldi, L. A. , Kitzberger, T. , & Chaneton, E. J. (2011). Environmental and genetic control of insect abundance and herbivory along a forest elevational gradient. Oecologia, 167(1), 117–129. 10.1007/s00442-011-1978-0 21476033

[ece36074-bib-0024] Gouinguené, S. P. , & Turlings, T. C. J. (2002). The effects of abiotic factors on induced volatile emissions in corn plants. Plant Physiology, 129(3), 1296–1307. 10.1104/pp.001941 12114583 PMC166523

[ece36074-bib-0025] Green, R. M. , & Tobin, E. M. (1999). Loss of the circadian clock‐associated protein 1 in Arabidopsis results in altered clock‐regulated gene expression. Proceedings of the National Academy of Sciences, 96(7), 4176–4179. 10.1073/pnas.96.7.4176 PMC22440 10097183

[ece36074-bib-0026] Guo, H. , Sun, Y. , Ren, Q. , Zhu‐Salzman, K. , Kang, L. E. , Wang, C. , … Ge, F. (2012). Elevated CO2 reduces the resistance and tolerance of tomato plants to *Helicoverpa armigera* by suppressing the JA signaling pathway. PLoS ONE, 7(7), e41426 10.1371/journal.pone.0041426 22829948 PMC3400665

[ece36074-bib-0027] Haukioja, E. , Suomela, J. , & Neuvonen, S. (1985). Long‐term inducible resistance in birch foliage: Triggering cues and efficacy on a defoliator. Oecologia, 65(3), 363–369. 10.1007/BF00378910 28310440

[ece36074-bib-0028] Hegland, S. J. , Jongejans, E. , & Rydgren, K. (2010). Investigating the interaction between ungulate grazing and resource effects on *Vaccinium myrtillus* populations with integral projection models. Oecologia, 163(3), 695–706. 10.1007/s00442-010-1616-2 20499103

[ece36074-bib-0029] Hegland, S. J. , Nielsen, A. , Lázaro, A. , Bjerknes, A.‐L. , & Totland, Ø. (2009). How does climate warming affect plant‐pollinator interactions? Ecology Letters, 12(2), 184–195. 10.1111/j.1461-0248.2008.01269.x 19049509

[ece36074-bib-0030] Hegland, S. J. , Seldal, T. , Lilleeng, M. S. , & Rydgren, K. (2016). Can browsing by deer in winter induce defence responses in bilberry (*Vaccinium myrtillus*)? Ecological Research, 31(3), 441–448. 10.1007/s11284-016-1351-1

[ece36074-bib-0031] Hjältén, J. , Danell, K. , & Ericson, L. (2004). Hare and vole browsing preferences during winter. Acta Theriologica, 49(1), 53–62. 10.1007/BF03192508

[ece36074-bib-0032] Hoffmann, L. , Besseau, S. , Geoffroy, P. , Ritzenthaler, C. , Meyer, D. , Lapierre, C. , … Legrand, M. (2004). Silencing of hydroxycinnamoyl‐coenzyme A shikimate/quinate hydroxycinnamoyltransferase affects phenylpropanoid biosynthesis. The Plant Cell, 16(6), 1446–1465. 10.1105/tpc.020297 15161961 PMC490038

[ece36074-bib-0033] IPCC (2018). IPCC report 2018: Global warming of 1.5°C: an IPCC special report on the impacts of global warming of 1.5°C above pre‐industrial levels and related global greenhouse gas emission pathways, in the context of strengthening the global response to the threat of climate change, sustainable development, and efforts to eradicate poverty. In IPCC Report.

[ece36074-bib-0034] Jacquemart, A.‐L. (1993). Floral visitors of *Vaccinium* species in the High Ardennes, Belgium. Flora, 188, 263–273. 10.1016/S0367-2530(17)32276-4

[ece36074-bib-0035] Jacquemart, A.‐L. , & Thompson, J. D. (1996). Floral and pollination biology of three sympatric *Vaccinium* (Ericaceae) species in the Upper Ardennes, Belgium. Canadian Journal of Botany, 74(2), 210–221.

[ece36074-bib-0036] Karban, R. , & Baldwin, I. T. (1997). Induced Responses to Herbivory. Chicago, IL: University of Chicago Press.

[ece36074-bib-0037] Karban, R. , Yang, L. H. , & Edwards, K. F. (2014). Volatile communication between plants that affects herbivory: A meta‐analysis. Ecology Letters, 17(1), 44–52. 10.1111/ele.12205 24165497

[ece36074-bib-0038] Körner, C. (2007). The use of ‘altitude’ in ecological research. Trends in Ecology & Evolution, 22(11), 569–574. 10.1016/j.tree.2007.09.006 17988759

[ece36074-bib-0039] Lattanzio, V. , Lattanzio, V. M. T. , & Cardinali, A. (2006). Role of phenolics in the resistance mechanisms of plants against fungal pathogens and insects. Phytochemistry: Advances in Research, 661, 23–67.

[ece36074-bib-0040] Li, P. , Ainsworth, E. A. , Leakey, A. D. B. , Ulanov, A. , Lozovaya, V. , Ort, D. R. , & Bohnert, H. J. (2008). Arabidopsis transcript and metabolite profiles: Ecotype‐specific responses to open‐air elevated [CO2]. Plant, Cell & Environment, 31(11), 1673–1687. 10.1111/j.1365-3040.2008.01874.x 18721265

[ece36074-bib-0041] Liland, K. H. , & Sæbø, S. (2014). mixlm: Mixed model ANOVA and statistics for education. R Package Version 1.7.

[ece36074-bib-0042] Makino, A. , Nakano, H. , Mae, T. , Shimada, T. , & Yamamoto, N. (2000). Photosynthesis, plant growth and N allocation in transgenic rice plants with decreased Rubisco under CO2 enrichment. Journal of Experimental Botany, 51:383–389. 10938846 10.1093/jexbot/51.suppl_1.383

[ece36074-bib-0043] Makino, A. , Sakuma, H. , Sudo, E. , & Mae, T. (2003). Differences between maize and rice in N‐use efficiency for photosynthesis and protein allocation. Plant and Cell Physiology, 44(9), 952–956. 10.1093/pcp/pcg113 14519777

[ece36074-bib-0044] Mizoguchi, T. , Wheatley, K. , Hanzawa, Y. , Wright, L. , Mizoguchi, M. , Song, H.‐R. , … Coupland, G. (2002). LHY and CCA1 are partially redundant genes required to maintain circadian rhythms in Arabidopsis. Developmental Cell, 2(5), 629–641. 10.1016/S1534-5807(02)00170-3 12015970

[ece36074-bib-0045] Moen, A. (1999). National atlas of Norway: Vegetation. Hønefoss, Norway: Norwegian Mapping Authority.

[ece36074-bib-0046] Moreira, X. , Mooney, K. A. , Rasmann, S. , Petry, W. K. , Carrillo‐Gavilán, A. , Zas, R. , & Sampedro, L. (2014). Trade‐offs between constitutive and induced defences drive geographical and climatic clines in pine chemical defences. Ecology Letters, 17(5), 537–546. 10.1111/ele.12253 24818235

[ece36074-bib-0047] Moreira, X. , Petry, W. K. , Mooney, K. A. , Rasmann, S. , & Abdala‐Roberts, L. (2018). Elevational gradients in plant defences and insect herbivory: recent advances in the field and prospects for future research. Ecography, 41(9), 1485–1496. 10.1111/ecog.03184

[ece36074-bib-0048] Mosolov, V. V. , & Valueva, T. A. (2011). Inhibitors of proteolytic enzymes under abiotic stresses in plants. Applied Biochemistry and Microbiology, 47(5), 453. 22232890

[ece36074-bib-0049] Nabity, P. D. , Zavala, J. A. , & DeLucia, E. H. (2013). Herbivore induction of jasmonic acid and chemical defences reduce photosynthesis in *Nicotiana attenuata* . Journal of Experimental Botany, 64(2), 685–694. 10.1093/jxb/ers364 23264519 PMC3542056

[ece36074-bib-0050] Norwegian Environment Agency . (2017). Retrieved from https://www.environment.no/topics/biodiversity/species-in-norway/deer/Rapport

[ece36074-bib-0051] Nunes‐Nesi, A. , Fernie, A. R. , & Stitt, M. (2010). Metabolic and signaling aspects underpinning the regulation of plant carbon nitrogen interactions. Molecular Plant, 3(6), 973–996. 10.1093/mp/ssq049 20926550

[ece36074-bib-0052] O'Connor, M. I. (2009). Warming strengthens an herbivore–plant interaction. Ecology, 90(2), 388–398. 10.1890/08-0034.1 19323223

[ece36074-bib-0053] Oleksiak, M. F. , Churchill, G. A. , & Crawford, D. L. (2002). Variation in gene expression within and among natural populations. Nature Genetics, 32(2), 261–266. 10.1038/ng983 12219088

[ece36074-bib-0054] Pearse, I. S. , & Hipp, A. L. (2012). Global patterns of leaf defenses in oak species. Evolution, 66(7), 2272–2286. 10.1111/j.1558-5646.2012.01591.x. 22759301

[ece36074-bib-0055] Pellissier, L. , Fiedler, K. , Ndribe, C. , Dubuis, A. , Pradervand, J.‐N. , Guisan, A. , & Rasmann, S. (2012). Shifts in species richness, herbivore specialization, and plant resistance along elevation gradients. Ecology and Evolution, 2(8), 1818–1825. 10.1002/ece3.296 22957184 PMC3433986

[ece36074-bib-0056] Pellissier, L. , Moreira, X. , Danner, H. , Serrano, M. , Salamin, N. , Dam, N. M. , & Rasmann, S. (2016). The simultaneous inducibility of phytochemicals related to plant direct and indirect defences against herbivores is stronger at low elevation. Journal of Ecology, 104(4), 1116–1125. 10.1111/1365-2745.12580

[ece36074-bib-0057] Post, E. , Forchhammer, M. C. , Syndonia Bret‐Harte, M. , Callaghan, T. V. , Christensen, T. R. , Elberling, B. , … Høye, T. T. (2009). Ecological dynamics across the arctic associated with recent climate change. Science, 325(5946), 1355–1358. 10.1126/science.1173113 19745143

[ece36074-bib-0058] Pratt, J. D. , & Mooney, K. A. (2013). Clinal adaptation and adaptive plasticity in *Artemisia californica*: Implications for the response of a foundation species to predicted climate change. Global Change Biology, 19(8), 2454–2466. 10.1111/gcb.12199 23505064

[ece36074-bib-0059] R Core Team . (2016). R: A language and environment for statistical computing. Vienna, Austria: R Foundation for Statistical Computing.

[ece36074-bib-0060] Rasmann, S. , Alvarez, N. , & Pellissier, L. (2014). The altitudinal niche‐breadth hypothesis in insect‐plant interactions. Annual Plant Reviews, 47, 339–359.

[ece36074-bib-0061] Rasmann, S. , Pellissier, L. , Defossez, E. , Jactel, H. , & Kunstler, G. (2014). Climate‐driven change in plant‐insect interactions along elevation gradients. Functional Ecology, 28(1), 46–54. 10.1111/1365-2435.12135

[ece36074-bib-0062] Rhoades, D. F. (1979). Evolution of plant chemical defense against herbi‐vores In Rosenthal G. A. A. J. D. H. (Ed.), Herbivores: Their interaction with secondary plant metabolites (pp. 3–54). Burlington, MA: Academic Press.

[ece36074-bib-0063] Ritchie, J. C. (1956). *Vaccinium myrtillus* L. Journal of Ecology, 44(1), 291–299. 10.2307/2257181

[ece36074-bib-0064] Rodríguez, A. A. , Maiale, S. J. , Menéndez, A. B. , & Ruiz, O. A. (2009). Polyamine oxidase activity contributes to sustain maize leaf elongation under saline stress. Journal of Experimental Botany, 60(15), 4249–4262. 10.1093/jxb/erp256 19717530

[ece36074-bib-0065] Rodriguez‐Saona, C. , Polashock, J. , & Malo, E. (2013). Jasmonate‐mediated induced volatiles in the American cranberry, *Vaccinium macrocarpon*: From gene expression to organismal interactions. Frontiers in Plant Science, 4, 115. 23641249 10.3389/fpls.2013.00115 PMC3638147

[ece36074-bib-0066] Schadt, E. E. , Monks, S. A. , Drake, T. A. , Lusis, A. J. , Che, N. , Colinayo, V. , … Friend, S. H. (2003). Genetics of gene expression surveyed in maize, mouse and man. Nature, 422(6929), 297 10.1038/nature01434 12646919

[ece36074-bib-0067] Schemske, D. W. , Mittelbach, G. G. , Cornell, H. V. , Sobel, J. M. , & Roy, K. (2009). Is there a latitudinal gradient in the importance of biotic interactions? Annual Review of Ecology Evolution and Systematics, 40, 245–269. 10.1146/annurev.ecolsys.39.110707.173430

[ece36074-bib-0068] Selas, V. (2001). Autumn population size of capercaillie Tetrao urogallus in relation to bilberry *Vaccinium myrtillus* production and weather: An analysis of Norwegian game reports. Wildlife Biology, 7(1), 17–25.

[ece36074-bib-0069] Seldal, T. , Hegland, S. J. , Rydgren, K. , Rodriguez‐Saona, C. , & Töpper, J. P. (2017). How to induce defense responses in wild plant populations? Using bilberry (*Vaccinium myrtillus*) as example. Ecology and Evolution, 7(6), 1762–1769. 28331586 10.1002/ece3.2687 PMC5355179

[ece36074-bib-0070] Serreze, M. C. , Walsh, J. E. , Chapin, F. S. , Osterkamp, T. , Dyurgerov, M. , Romanovsky, V. , … Barry, R. G. (2000). Observational evidence of recent change in the northern high‐latitude environment. Climatic Change, 46(1–2), 159–207.

[ece36074-bib-0071] Springer, C. J. , Orozco, R. A. , Kelly, J. K. , & Ward, J. K. (2008). Elevated CO2 influences the expression of floral‐initiation genes in *Arabidopsis thaliana* . New Phytologist, 178(1), 63–67. 18315697 10.1111/j.1469-8137.2008.02387.x

[ece36074-bib-0072] Sun, Y. , Yin, J. , Cao, H. , Li, C. , Kang, L. E. , & Ge, F. (2011). Elevated CO2 influences nematode‐induced defense responses of tomato genotypes differing in the JA pathway. PLoS ONE, 6(5), e19751 10.1371/journal.pone.0019751 21629688 PMC3101209

[ece36074-bib-0073] Trussell, G. C. , David, L. , & Smith, L. D. (2000). Induced defenses in response to an invading crab predator: An explanation of historical and geographic phenotypic change. Proceedings of the National Academy of Sciences, 97(5), 2123–2127. 10.1073/pnas.040423397 PMC15764 10681425

[ece36074-bib-0074] Van Dam, N. M. , & Baldwin, I. T. (2001). Competition mediates costs of jasmonate‐induced defences, nitrogen acquisition and transgenerational plasticity in *Nicotiana * *attenuata* . Functional Ecology, 15(3):406–415.

[ece36074-bib-0075] Velásquez, A. C. , Castroverde, C. D. M. , & He, S. Y. (2018). Plant–pathogen warfare under changing climate conditions. Current Biology, 28(10), R619–R634. 10.1016/j.cub.2018.03.054 29787730 PMC5967643

[ece36074-bib-0076] Veteli, T. O. , Kuokkanen, K. , Julkunen‐Tiito, R. , Roininen, H. , & Tahvanainen, J. (2002). Effects of elevated CO2 and temperature on plant growth and herbivore defensive chemistry. Global Change Biology, 8(12), 1240–1252.

[ece36074-bib-0077] Winning, H. , Viereck, N. , Wollenweber, B. , Larsen, F. H. , Jacobsen, S. , Søndergaard, I. B. , & Engelsen, S. B. (2009). Exploring abiotic stress on asynchronous protein metabolism in single kernels of wheat studied by NMR spectroscopy and chemometrics. Journal of Experimental Botany, 60(1), 291–300. 10.1093/jxb/ern293 19213725 PMC3071774

[ece36074-bib-0078] Yang, S. , Huihui, W. , Xie, J. , & Rantala, M. J. (2013). Depressed performance and detoxification enzyme activities of Helicoverpa armigera fed with conventional cotton foliage subjected to methyl jasmonate exposure. Entomologia Experimentalis Et Applicata, 147(2):186–195.

[ece36074-bib-0079] Zeller, G. , Henz, S. R. , Widmer, C. K. , Sachsenberg, T. , Rätsch, G. , Weigel, D. , & Laubinger, S. (2009). Stress‐induced changes in the *Arabidopsis thaliana* transcriptome analyzed using whole‐genome tiling arrays. The Plant Journal, 58(6), 1068–1082. 19222804 10.1111/j.1365-313X.2009.03835.x

[ece36074-bib-0080] Zobayed, S. M. A. , Afreen, F. , & Kozai, T. (2005). Temperature stress can alter the photosynthetic efficiency and secondary metabolite concentrations in St. John's wort. Plant Physiology and Biochemistry, 43(10–11), 977–984. 10.1016/j.plaphy.2005.07.013 16310362

[ece36074-bib-0081] Züst, T. , & Agrawal, A. A. (2017). Trade‐offs between plant growth and defense against insect herbivory: An emerging mechanistic synthesis. Annual Review of Plant Biology, 68, 513–534. 10.1146/annurev-arplant-042916-040856 28142282

