# Supplementary material for: Molecular and ecological plant defense responses along an elevational gradient in a boreal ecosystem
Source: Ecol Evol. 2020 Feb 5;10(5):2478–91. doi: 10.1002/ece3.6074 (PMC7069305; doi:10.1002/ece3.6074)
Supplement: Supplementary file 1 [file ECE3-10-2478-s001.docx]

**Supplementary Information**

**S1. Target genes and primer sequences used for real-time qPCR.**

| Gene (*abbreviation*) | F Primer | R Primer |
| --- | --- | --- |
| Shikimate O-hydroxycinnamoyltransferase (*SHIKIMATE*) | TCTTCACCGGCACATCATTAG | CGTCGTCCATTCGGGATAAA |
| Tyrosine aminotransferase (*TYR*) | GCCCATTCCGAGAGAAATCA | CCGAACACCATCACAATCAAAG |
| Leucoanthocyanidin dioxygenase (*LDOX*); | TGTAGAAGAAGGCAAGCGATAC | CGGCGATCAAATTCAGGTACTA |
| UDP-glycosyltransferase (*UDP*); | CAACCGCAGTTGCCATAAAC | GTGGTGGTGGTGGTGATATT |
| MYB-related transcription factor LHY (*LHY*); | GTTCGCCAGTCTACCACTAAA | CGAGGAGAGAGATGGAGGATTA |
| Flavonoid 3',5'-hydroxylase (*FLAV*); | GACCTCGGCTGGACTATTTAAC | CATACGCTTCATCCCACTCTC |
| Photosystem II PsbW (*PHOTO*); | CAAGGGAGATCCACAGCATATT | CAAGGCAAACCCATGGAAATAC |
| Glutamine synthetase chloroplastic (*GLU*). | AGCCACCATCCTCTCTCATA | GTGTTGTACTCACTCTCGATCC |
| Fbox protein (*FBOX*) | AATTCATAATCCCAGCCCCTC | TCCGTCCACAAAAGAGTCATC |
| Glyceraldehyde 3-phosphate dehydrogenase (*GAP*). | GAAGGATTGGAGAGGTGGAAG | CCACAGTAGGAACACGGAAC |
| Ubiquitin-conjugating enzyme E2 28 (*UBC28*) | GATGAACCTCACCAAAATACCTG | ACCCCGCTCTCAATCATAAC |

| **Number of leaves** | 26.14 | <0.0001*** | - | - | - | - | - | - |
| --- | --- | --- | --- | --- | --- | --- | --- | --- |
| **Number of shoots** | - | - | 8.43 | <0.05* | - | - | - | - |
| **Dry mass** | - | - | - | - | 13.81 | <0.0001*** | - | - |
| **Flowers** | - | - | - | - | - | - | 1.31 | 0.25 |

**S2. ANOVA supplementary table with Chi-square and p-value for seasonal changes in insect and mammalian herbivory, growth (dry mass) and reproduction (fruit set).** Significance codes: ‘***’ < 0.0001; ‘**’ < 0.001; ‘*’ < 0.05; ‘°’ < 0.1.

| Response variables | **Insect herbivory** | **Mammalian herbivory** | **Growth (dry mass)** | **Reproduction (fruit set)** |
| --- | --- | --- | --- | --- |

| Factors | **Chisq** | **P-value** | **Chisq** | **P-value** | **Chisq** | **P-value** | **Chisq** | **P-value** |
| --- | --- | --- | --- | --- | --- | --- | --- | --- |

| **Treat** | 42.22 | <0.0001*** | 10.14 | <0.001** | 7.76 | <0.005* | 91.25 | <0.0001*** |
| --- | --- | --- | --- | --- | --- | --- | --- | --- |
| **Site** | 0.51 | 0.77 | 7.05 | <0.05* | 12.26 | <0.05* | 9.63 | <0.05* |

| Covariates |  |  |  |  |  |  |  |  |
| --- | --- | --- | --- | --- | --- | --- | --- | --- |

| **Year** | 0.25 | 0.61 | 0.01 | 0.93 | 8.16 | <0.05* | 8.63 | <0.05* |
| --- | --- | --- | --- | --- | --- | --- | --- | --- |

| Interactions |  |  |  |  |  |  |  |  |
| --- | --- | --- | --- | --- | --- | --- | --- | --- |

| **Treat:Site** | 7.14 | <0.05* | 0.16 | 0.92 | 2.51 | 0.28 | 30.85 | <0.0001*** |
| --- | --- | --- | --- | --- | --- | --- | --- | --- |

| **Treat:Year** | 8.53 | <0.05* | 0.10 | 0.75 | 0.01 | 0.93 | 11.09 | <0.001** |
| --- | --- | --- | --- | --- | --- | --- | --- | --- |
| **Treat:Site:Year** | 12.04 | <0.05* | 9.21 | <0.05* | 13.54 | <0.05* | 16.21 | <0.05* |

**S3. ANOVA supplementary table with F and p-values for gene expression levels of defense and growth-related target genes.** Significance codes: ‘***’ < 0; ‘**’ < 0.001; ‘*’ < 0.05; ‘°’ < 0.1.

| Genes | **FLAV** | | **TYR** | | **LHY** | | **SHIKIMATE** | | **LDOX** | | **UDP** | | **GLU** | | **PHOTO** | |
| --- | --- | --- | --- | --- | --- | --- | --- | --- | --- | --- | --- | --- | --- | --- | --- | --- |
| Factors | **F** | **P-value** | **F** | **P-value** | **F** | **P-value** | **F** | **P-value** | **F** | **P-value** | **F** | **P-value** | **F** | **P-value** | **F** | **P-value** |
| **Treatment** | 49.33 | <0.0001*** | 38.79 | <0.0001*** | 3.76 | <0.1° | 85.49 | <0.0001*** | 74.65 | <0.0001*** | 14.2 | <0.0001*** | 21.26 | <0.0001*** | 5.54 | <0.05* |
| **Site** | 47.99 | <0.0001*** | 2.25 | 0.12 | 20.68 | <0.0001*** | 63.99 | <0.0001*** | 32.5 | <0.0001*** | 20.4 | <0.0001*** | 96.5 | <0.0001*** | 26.17 | <0.0001*** |
| **Year** | 73.3 | <0.0001*** | 205.5 | <0.0001*** | 141.6 | <0.0001*** | 112.25 | <0.0001*** | 165.6 | <0.0001*** | 15.4 | <0.0001*** | 442.68 | <0.0001*** | 35.98 | <0.0001*** |
| Interactions |  |  |  |  |  |  |  |  |  |  |  |  |  |  |  |  |
| **Treat:Site** | 2.78 | <0.1° | 1.2 | 0.31 | 0.19 | 0.82 | 5.33 | <0.05* | 6.43 | <0.001** | 2.37 | 0.11 | 14.05 | <0.0001*** | 1.42 | 0.26 |
| **Treat:Year** | 42.67 | <0.0001*** | 25.16 | <0.0001*** | 6.75 | <0.05* | 4.98 | <0.05* | 17.33 | <0.0001*** | 11.23 | <0.001** | 6.4 | <0.05* | 22.5 | <0.0001*** |
| **Site:Year** | 6.57 | <0.05* | 0.05 | 0.94 | 1.97 | 0.16 | 9.55 | <0.0001*** | 7.02 | <0.001** | 5.66 | <0.001** | 32.84 | <0.0001*** | 0.11 | 0.89 |
| **Treat:Site:Year** | 5.81 | <0.05* | 4.66 | <0.05* | 0.36 | 0.69 | 3.11 | <0.1° | 0.86 | 0.43 | 4.33 | <0.05* | 3.57 | <0.05* | 6.79 | <0.001** |
